# Supplementary material for: Improvements in Body Composition after a Proposed Anti-Inflammatory Diet Are Modified by Employment Status in Weight-Stable Patients with Rheumatoid Arthritis, a Randomized Controlled Crossover Trial
Source: Nutrients. 2022 Mar 2;14(5):1058. doi: 10.3390/nu14051058 (PMC8912542; doi:10.3390/nu14051058)
Supplement: Supplementary file 1 [file nutrients-14-01058-s001.zip › nutrients-1589914-supplementary.pdf]

Table S1. Modelled estimates of differences in body composition, and metabolic markers hypothetically related to body composition, between intervention and control diet periods among patients with RA in the ADIRA trial [1].

	All Participants (n = 42)			
	**Intervention**	**Control**	**Difference between Periods**	
	Mean (95% CI)	Mean (95% CI)	Mean (95% CI)	P [2]
**Measures of body composition**				
Weight (kg)	-0.282 (-0.833, 0.269)	0.136 (-0.414, 0.687)	-0.419 (-1.198, 0.361)	0.288
BMI (kg/m^2)	-0.090 (-0.286, 0.106)	0.033 (-0.162, 0.229)	-0.123 (-0.400, 0.154)	0.379
FFM (kg)	0.935 (0.278, 1.591)	0.778 (0.123, 1.434)	0.156 (-0.677, 0.989)	0.706
FFMI (kg/m^2)	0.324 (0.102, 0.545)	0.258 (0.037, 0.479)	0.066 (-0.212, 0.343)	0.635
FM (kg)	-0.995 (-1.889, -0.101)	-0.786 (-1.680, 0.107)	-0.208 (-1.474, 1.057)	0.744
FMI (kg/m^2)	-0.346 (-0.642, -0.050)	-0.251 (-0.547, 0.044)	-0.094 (-0.514, 0.325)	0.655
FM (%)	-1.364 (-2.251, -0.476)	-1.219 (-2.105, -0.333)	-0.144 (-1.401, 1.112)	0.820
		**Biomarkers**		
BCAA [3] (mmol/L)	-0.016 (-0.034, 0.001)	0.005 (-0.013, 0.022)	**-0.021 (-0.041, -0.001)**	**0.042**
IGF-1 (µg/L)	-0.642 (-6.951, 5.667)	4.388 (-1.913, 10.689)	-5.030 (-13.954, 3.894)	0.265

BCAA, Branched chain amino acids; BMI, Body mass index; FFM, Fat free mass; FFMI, Fat free mass index; FM, Fat mass; FMI, Fat mass index; IGF-1, Insulin-like growth factor 1.

[1] Participants completing at least one diet period and where measurement of body composition was possible (n = 42). RA, Rheumatoid Arthritis; ADIRA, Anti-inflammatory Diet In Rheumatoid Arthritis. Analysed by use of a linear mixed model with period, treatment, sex and baseline value as fixed effects and subject as random effect.

[2] Intervention – Control values.

[3] Leucine, isoleucine and valine combined.

Table S2. Baseline data of participants who completed at least one diet period, stratified by employment status.

	**Not Employed (n = 15)**	**Employed (n = 27)**	**P**
	**Median (IQR)**	**Median (IQR)**	
Age (year)	70.8 (69.5, 72.6)	58.7 (45.9, 62.6)	**< 0.001** [a]
Disease duration (year)	21 (16.3, 29.9)	17.7 (9.7, 20.6)	0.105 [a]
Weight (kg)	75.5 (66.8, 85.35)	77.75 (65.7, 82.8)	0.810 [a]
BMI (kg/m^2)	29.9 (24.8, 32.3)	25.1 (22.9, 27.7)	0.093 [a]
FFMI (kg/m^2)	17.6 (15.1, 18.8)	17.5 (15.3, 18.4)	0.917 [a]
FMI (kg/m^2)	11.4 (9.2, 13.2)	7.9 (6.3, 10.9)	**0.013** [a]
FM (%)	39.5 (37.0, 43.0)	34.0 (27.6, 40.1)	**0.011** [a]
Waist-Hip ratio	0.83 (0.80, 0.88)	0.85 (0.79, 0.92)	0.736 [a]
DAS28	3.71 (3.11, 4.66)	3.37 (3.01, 4.65)	0.697 [a]
Erythrocyte Sedimentation Rate (mm/h)	20 (11, 29)	16 (9, 24)	0.352 [a]
C-reactive Protein (mg/L)	4 (2, 6)	2 (1, 4)	0.070 [a]
HAQ	0.88 (0.38, 1.25)	0.38 (0.13, 1.13)	0.104 [a]
Albumin (g/L)	37 (36, 39)	39 (38, 42)	0.063 [a]
IGF-1 (µg/L)	98 (80, 135)	121 (88, 160)	0.152 [a]
**Dietary intake**			
NRF11.3	7.1 (6.2, 8.5)	6.0 (4.7, 7.4)	**0.008** [a]
Dietary Quality Index	7 (6, 8)	6 (5, 7)	0.169 [a]
Energy (kcal)	1660 (1200, 2010)	1930 (1540, 2240)	0.174 [a]
Protein (E%)	15.5 (14.8, 19.8)	15.4 (13.6, 16.5)	0.420 [a]
Carbohydrate (E%) (including fiber)	44.1 (40.9, 51.7)	42.2 (38.0, 47.7)	0.362 [a]
Alcohol (E%)	0.00 (0.00, 0.78)	2.09 (0.00, 5.25)	0.117 [a]
Fat (E%)	35.5 (33.0, 37.0)	37.4 (31.7, 41.7)	0.285 [a]
Saturated fatty acids (E%)	13.7 (12.7, 15.0)	15.7 (12.5, 17.2)	0.310 [a]
Monounsaturated fatty acids (E%)	11.7 (11.0, 13.4)	13.0 (11.2, 16.5)	0.136 [a]
Polyunsaturated fatty acids (E%)	5.4 (5.2, 7.8)	5.0 (4.1, 7.4)	0.262 [a]
Marine omega-3 fatty acids [1] (E%)	0.32 (0.09, 0.96)	0.09 (0.06, 0.48)	**0.042** [a]
Protein (g/kg bodyweight/day)	0.88 (0.66, 1.09)	0.9 (0.82, 1.2)	0.499 [a]
Protein (g/day)	66 (54, 74)	70 (56, 84)	0.402 [a]
Fiber (g/day)	18 (15, 21)	18 (14, 21)	0.626 [a]
Meal frequency (meals per day > 25 kcal)	5.0 (4.0, 5.3)	5.0 (4.0, 5.3)	0.891 [a]
	**n (%)**	**n (%)**	
**Medication**			
bDMARD usage	5 (33.3)	12 (44)	0.531 [b]
csDMARD usage	8 (53.5)	24 (89)	**0.020** [b]
**Measures of rheumatoid cachexia**			
Engvall [2]	2 (13)	5 (19)	1.000 [b]
Elkan [3]	2 (13)	10 (37)	0.158 [b]
High FMI [4]	9 (60)	14 (52)	0.750 [b]
Low FFMI [5]	4 (27)	11 (41)	0.506 [b]
Low FFMI & high FMI [6]	1 (7)	8 (30)	0.123 [b]
**Sex**			
Female	15 (100)	19 (70)	**0.035** [b]
**Parental origin**			0.439 [b]
Europe	13 (87)	26 (96)	
Africa	1 (7)	0 (0)	
Asia	1 (7)	1 (4)	
**Smokers**	0 (0)	2 (7)	0.530 [b]
**Educational level**			0.432 [b]
Junior high school	3 (20)	2 (7)	
2 year senior high school	4 (27)	4 (15)	
≥3 year senior high school	2 (13)	4 (15)	
College or university	6 (40)	17 (63)	
**Physical activity during everyday life**			0.519 [b]
Light	1 (7)	4 (15)	
Light but partly active	6 (40)	8 (30)	
Light and active	6 (40)	7 (26)	
Sometimes physically heavy	2 (13)	8 (30)	
Physically heavy most of the time	0 (0)	0 (0)	
**Intentional physical exercise**			0.890 [b]
Never	3 (20)	3 (11)	
Now and then, not regularly	5 (33)	8 (30)	
1-2 times/week	2 (13)	7 (26)	
2-3 times/week	3 (20)	5 (19)	
>3 times/week	2 (13)	4 (15)	

bDMARD, biological disease modifying anti-rheumatic drug; BMI, Body mass index; csDMARD, conventional synthetic disease modifying anti-rheumatic drug; DAS28, Disease Activity Score 28 joints;

FFMI, Fat free mass index; FM, Fat mass; FMI, Fat mass index; HAQ, Health assessment questionnaire disability index; Insulin-like growth factor 1, IGF-1; NRF11.3, Nutrient Rich Foods index 11.3.

[1] docosahexaenoic, docosapentaenoic and eicosapentaenoic acid combined.
[2] Defined as FFMI $<10^{th}$ percentile and FMI $>25^{th}$ percentile as proposed by Engvall et al. [19].
[3] Defined as FFMI $<25^{th}$ percentile and FMI $>50^{th}$ percentile as proposed by Elkan et al. [20].
[4] Defined as FMI $>75^{th}$ percentile.
[5] Defined as FFMI $<25^{th}$ percentile.
[6] Defined as FFMI $<25^{th}$ and FMI $>75^{th}$ percentile.
Reference for body composition ranges is taken from Schutz et al. [21].

[a] Mann-Whitney Test using the exact *p*-value.
[b] Fisher's exact test.
